# Supplementary material for: Splice-Junction-Based Mapping of Alternative Isoforms in the Human Proteome
Source: Cell Rep. Author manuscript; Available in PMC 2020 Jan 15. (PMC6961840; doi:10.1016/j.celrep.2019.11.026)
Supplement: 3 [file NIHMS1546469-supplement-3.zip › DF2/PXD0010154/Spleen-23-O95197-SCSSSCAVHDLIFWR.pdf]

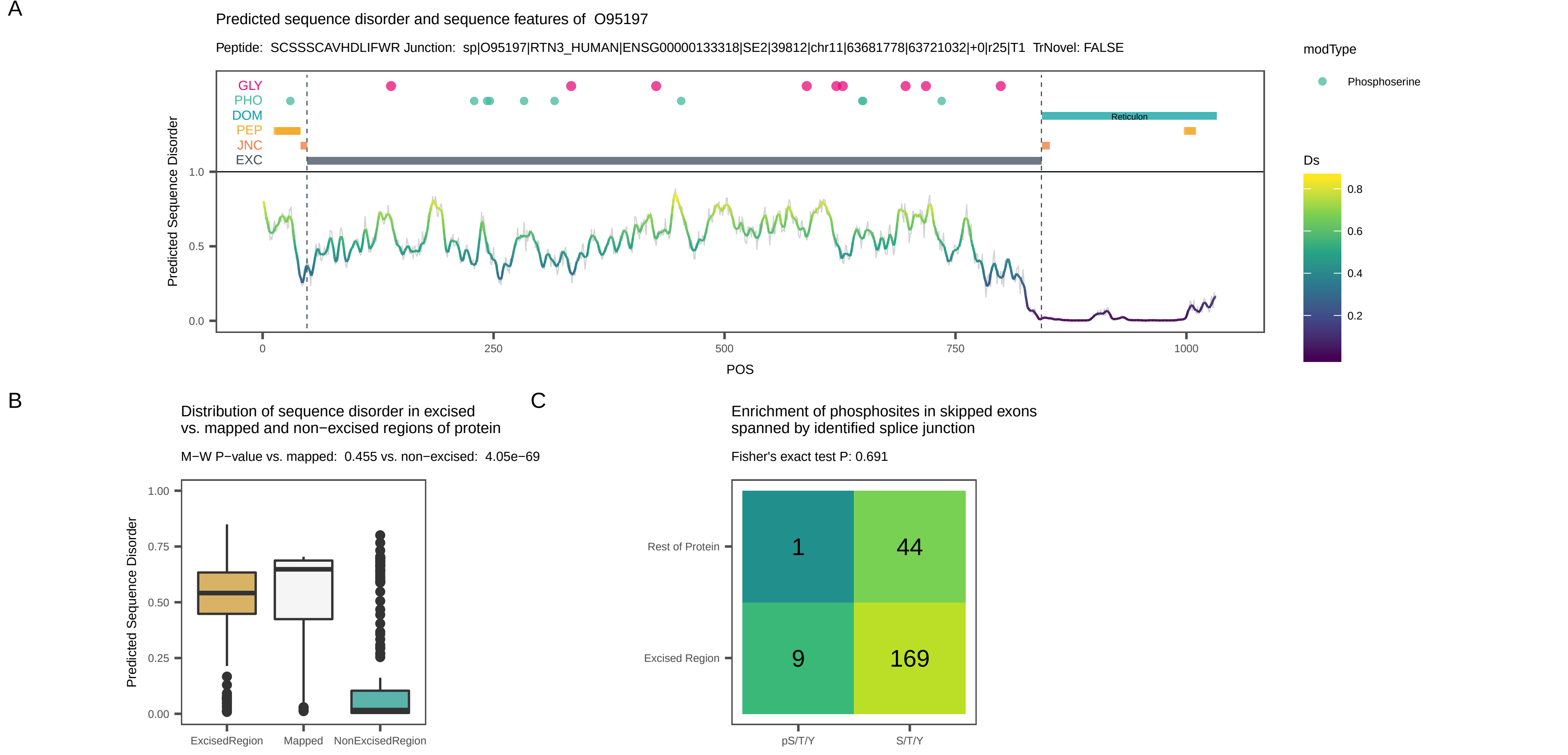

A
Predicted sequence disorder and sequence features of O95197
Peptide: SCSSSCAVHDLIFWR Junction: sp|O95197|RTN3_HUMAN|ENSG00000133318|SE2|39812|chr11|63681778|63721032|+0|r25|T1 TrNovel: FALSE
GLY
PHO
DOM
PEP
JNC
EXC
Reticulon
Predicted Sequence Disorder
1.0
0.5
0.0
0
250
500
750
1000
POS
modType
Phosphoserine
Ds
0.8
0.6
0.4
0.2
B
Distribution of sequence disorder in excised vs. mapped and non−excised regions of protein
M−W P−value vs. mapped: 0.455 vs. non−excised: 4.05e−69
Predicted Sequence Disorder
1.00
0.75
0.50
0.25
0.00
ExcisedRegion
Mapped
NonExcisedRegion
C
Enrichment of phosphosites in skipped exons spanned by identified splice junction
Fisher's exact test P: 0.691
Rest of Protein
Excised Region
1
44
9
169
pS/T/Y
S/T/Y
